# Health and well-being of mothers and co-parents during the first 12 months post partum: study protocol of the national SOCRATES cohort study in Switzerland

**DOI:** 10.1136/bmjopen-2026-121443

**Published:** 2026-06-04

**Authors:** Laurent Gaucher, Thomas Desplanches, Jessica Sormani, Gilles Cattani, Antonia N Mueller, Emilienne Celetta, Isabel N Widmer, Elsa Lorthe, Susanne Grylka-Baeschlin

**Affiliations:** 1 Geneva School of Health Sciences, HES-SO University of Applied Sciences and Arts Western Switzerland, Geneva, Switzerland; 2 INSERM, RESHAPE, UMR S1290, Université Lyon 1, Lyon, France; 3 Department of Women, Child and Adolescent, University Hospitals of Geneva, Geneva, Switzerland; 4 Research Institute for Midwifery and Reproductive Health, ZHAW Zurich University of Applied Sciences, Winterthur, Switzerland; 5 Centre for Research in Epidemiology and Statistic, Université Paris Cité and Université Sorbonne Paris Nord, Inserm, INRAE, Paris, France; 6 Midwifery Division, Bern University of Applied Sciences, Bern, Switzerland

**Keywords:** Postpartum Period, Parents, Quality of Life, MENTAL HEALTH, Maternal medicine

## Abstract

**Introduction:**

The first year after childbirth is a critical yet insufficiently monitored period for parental health. Postpartum mental and physical morbidity can affect both mothers and co-parents, but national longitudinal data remain scarce. The Stress Of Co-parents Related to A Traumatic Experience of birth across Switzerland (SOCRATES) cohort study aims to describe maternal and co-parental health and well-being trajectories during the first year after childbirth.

**Methods and analysis:**

SOCRATES is a prospective, population-based cohort study conducted in all linguistic regions of Switzerland. Eligible participants include women aged 14 and above who gave birth to a live or stillborn infant (≥22^+0^ weeks’ gestation and ≥500 g) and their cohabiting co-parents, provided they speak German, French, Italian or English. Recruitment was conducted in 81 of the 112 Swiss maternity units, birth centres and organisations of midwives over 6 weeks in spring 2025. Clinical data on pregnancy, childbirth and the early postpartum period are extracted from medical records. Postpartum hospitalisation data are obtained through linkage with national medico-administrative databases. Participants complete online questionnaires shortly after birth and at 2, 6 and 12 months post partum, including sociodemographic characteristics and patient-reported outcomes. The primary outcome is the prevalence of childbirth-related post-traumatic stress disorder at 2 months, assessed using the City Birth Trauma Scale. Secondary outcomes include depression, physical recovery, sexual health, quality of life, healthcare use, perceived care quality and overall well-being. A weighting procedure will be used to ensure representativeness and to account for attrition.

**Ethics and dissemination:**

Ethical approval was granted by all seven Swiss ethics committees (number 2024-02262). All participants provided informed consent. Findings will be disseminated through national and international conferences, peer-reviewed publications, policy briefs, social media and stakeholder engagement activities.

**Trial registration number:**

NCT06886841.

**STRENGTHS AND LIMITATIONS OF THIS STUDY**

- This is the first national prospective cohort in Switzerland to assess the health and well-being of both mothers and co-parents during the first year post partum, with national representativeness supported by a multistep weighting procedure to account for non-participation and attrition.
- The study combines clinical data, multilingual patient-reported outcomes, linkage to national hospital administrative databases, and institutional-level survey data from maternity units, enabling multilevel analyses of physical, mental, sexual and social health, care practices and overall well-being.
- The study takes a comprehensive approach by examining both adverse health outcomes and positive indicators such as well-being, parental bonding, flourishing and life satisfaction.
- As with most observational longitudinal studies, reliance on self-reported measures may introduce recall or reporting bias, and loss to follow-up may limit data completeness.

## Introduction

 The first year after childbirth is a critical yet underaddressed period for parental health and family well-being.^1 2^ Postpartum care and research have traditionally focused on the first weeks after birth and on selected outcomes, particularly depression and short-term physical morbidity. This focus has left important gaps in knowledge about longer-term recovery, sexual and social health, care experiences and the needs of co-parents.^1 2^ Many women report unresolved complications beyond the early postpartum period, including fatigue, pain, sexual dysfunction and psychological distress.^2^,^4^ These sequelae can affect individual well-being, family life, partner relationship and caregiving capacity. Addressing these needs requires moving beyond a fragmented approach. Postpartum health can be conceptualised as a multidimensional process shaped by biological, psychological and social factors, as well as by care experiences and broader social and structural determinants, consistent with the biopsychosocial model,^5^ the WHO frameworks on social determinants of health and the WHO framework for quality of maternal and newborn care.^15^,^8^ This perspective supports the study of multiple interrelated domains over time, including mental health, physical recovery, sexual health, care experiences, social context and co-parent health.

Within this multidimensional approach, mental health constitutes a central component of postpartum health. It represents a major clinical and public health concern, with potential consequences for parents, infants and family functioning.^9 10^ Depression and anxiety affect a substantial proportion of mothers and co-parents,^9^,^11^ and some develop childbirth-related post-traumatic stress disorder (CB-PTSD), a subtype of PTSD with specific perinatal triggers.^12^,^14^ In Switzerland, a monocentric study with a small convenience sample reported that 20.7% of mothers and 7.2% of fathers met criteria for probable PTSD 1 month post partum.^15^ Although the study relied on a general PTSD screening tool and was affected by selection bias, it highlighted the potential magnitude of trauma symptoms and the clinical relevance of avoidance behaviours. Yet, long-term support remains limited, and trajectories of recovery are rarely monitored, despite their known consequences on quality of life.^3 16^ Avoidance and disengagement from care, recognised as key features of CB-PTSD, may also compromise help-seeking and widen existing gaps in support.

The quality and experience of care during childbirth and the postpartum period represent another key component of this framework, as they may shape health trajectories across multiple domains.^7 17^ According to the WHO, high-quality maternity care should be safe, effective, timely, equitable and people-centred and should integrate both provision and experience of care.^7 8^ Clinical practices during childbirth, such as labour induction, episiotomy, caesarean delivery or limited access to pain relief, have been associated with trauma, dissatisfaction and poor mental health outcomes.^18^ In Switzerland, postpartum follow-up includes an average of 7.9 home visits per mother, one of the highest rates in Europe.^19^ However, little is known about how the content and perceived quality of this care relate to health trajectories after childbirth. Despite growing attention to respectful maternity care, few population-based studies investigate how care provision and care experiences relate to long-term postpartum recovery, particularly in interaction with mental health, physical recovery and broader social determinants.^2 17^

### Study aims

**Figure 1:**
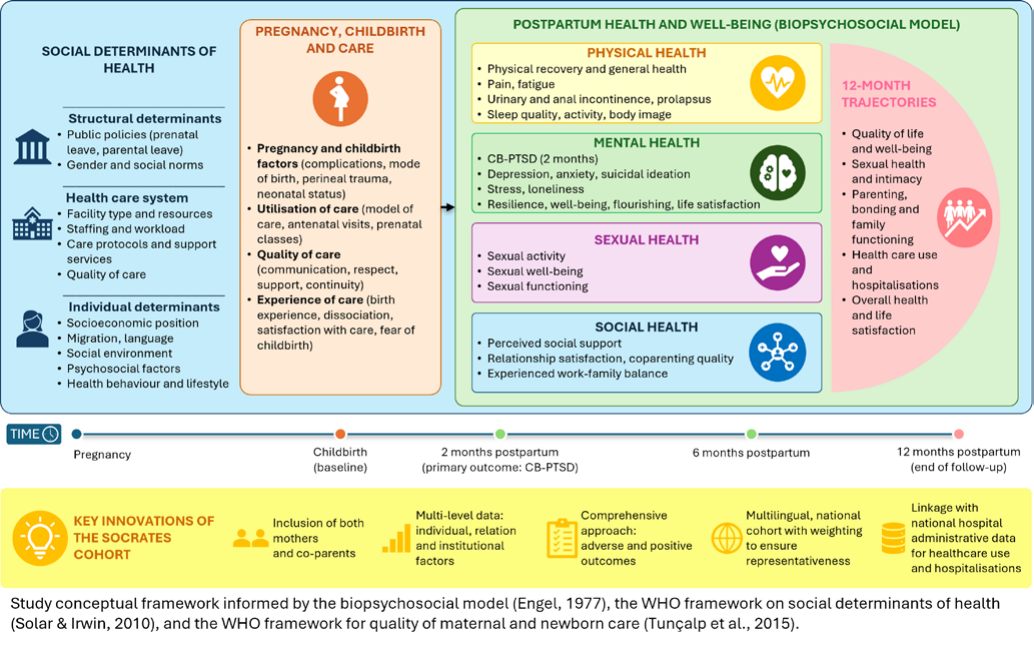
Study conceptual framework of postpartum health and well-being trajectories in the SOCRATES cohort. BW, birth weight; GA, gestational age; SOCRATES, Stress Of Co-parents Related to A Traumatic Experience of birth across Switzerland.

This study aims to examine the evolution of the health and well-being of both mothers and co-parents during the first year after childbirth, using a population-based longitudinal design. Guided by the conceptual framework presented in figure 1, the study integrates mental, physical, sexual and social health outcomes with clinical data, care experiences and determinants measured at individual, health-system and structural levels.^15^,^8^ The primary objective is to (1) estimate the prevalence of CB-PTSD in both mothers and co-parents at 2 months post partum. The secondary objectives are to (2) assess depressive and anxiety symptoms from the perinatal period to 12 months postpartum and identify their early predictors; (3) evaluate maternal recovery, including early postpartum recovery, fatigue, pain and incontinence, as well as general health status in both parents; (4) describe sexual health at 6 and 12 months including sexual dysfunction and well-being; (5) assess trajectories of health-related quality of life and well-being in both parents; (6) examine how clinical practices at birth (eg, induction, mode of childbirth, perineal trauma) and structural characteristics (eg, staffing levels, care protocols, availability of support services), together with the perceived quality of intrapartum care (eg, communication, support, respect) influence health outcomes; (7) estimate the frequency and causes of maternal and neonatal hospitalisations, using linkage with national hospital discharge data and (8) analyse social and gender-related inequalities in postpartum health and well-being.

## Methods and analysis

This protocol was developed in accordance with the Standard Protocol Items Recommendations for Observational Studies (SPIROS) checklist.^20^ The study was registered on ClinicalTrials.gov under the study acronym ‘SOCRATES’ (Stress Of Co-parents Related to A Traumatic Experience of birth across Switzerland, NCT06886841). The registration was submitted on 12 March 2025 and last updated on 20 March 2025.

### Study design and population

The SOCRATES study is a nationwide, population-based, prospective cohort study conducted in Switzerland. The study is coordinated through a national collaboration between university-affiliated research teams and academic hospital partners in all linguistic regions, with the support of professional societies (ie, Swiss Federation of Midwives) and patient advocacy organisations (ie, Santé sexuelle Suisse and Periparto).

The study population comprises mothers and co-parents following childbirth. The term ‘mother’ is used solely to distinguish birthing individuals from their partners; any birthing person who meets the inclusion criteria, regardless of gender identity, is eligible. Mothers are eligible if they are aged 14 years or older and have given birth to one or more liveborn or stillborn infants at a gestational age of ≥22^+0^ weeks and with a birthweight ≥500 g. Co-parents are eligible if they are aged 14 years or older, their partner agrees to participate in the study and they are living together. As all study materials are provided in four languages (German, French, Italian and English), eligibility requires sufficient proficiency in one of these languages to ensure informed consent and valid questionnaire responses. Exclusion criteria are limited to refusal to participate or inability to provide informed consent.

### Sample size and power calculation

The required sample size was determined a priori based on the primary objective of estimating the prevalence of CB-PTSD in mothers at 2 months post partum. Assuming a prevalence of 5% in high-income countries^21^ and aiming for a 95% CI with a ±1% margin of error, we estimated that at least 1900 complete responses at 2 months would be needed. Anticipating a 60% follow-up rate, based on previous large-scale perinatal cohorts,^22 23^ we set a baseline recruitment target of approximately 3200 women. This sample size also allows for planned subgroup analyses on care practices.^24^

### Recruitment and participation

Recruitment was conducted across 81 maternity units and birth centres, covering all linguistic regions and representing 72% of the 112 Swiss birth facilities. All centres recruited for a total of 6 weeks, with most conducting recruitment between 17 March and 27 April 2025. In some centres, recruitment periods were staggered due to local organisational constraints; however, all recruitment activities were completed by 11 June 2025. In each centre, a local investigator coordinated recruitment and data collection. All women giving birth during the recruitment period were systematically screened for eligibility. Eligible women received oral and written information after childbirth and were invited to provide electronic informed consent for (1) extraction of clinical data from medical records and completion of online questionnaires (with the option to consent to clinical data extraction only), (2) data linkage with hospital discharge records and (3) optional recontact after the first year for future research participation. Eligible co-parents were invited following the same procedures, except that consent for access to medical data and data linkage was not required.

**Figure 2:**
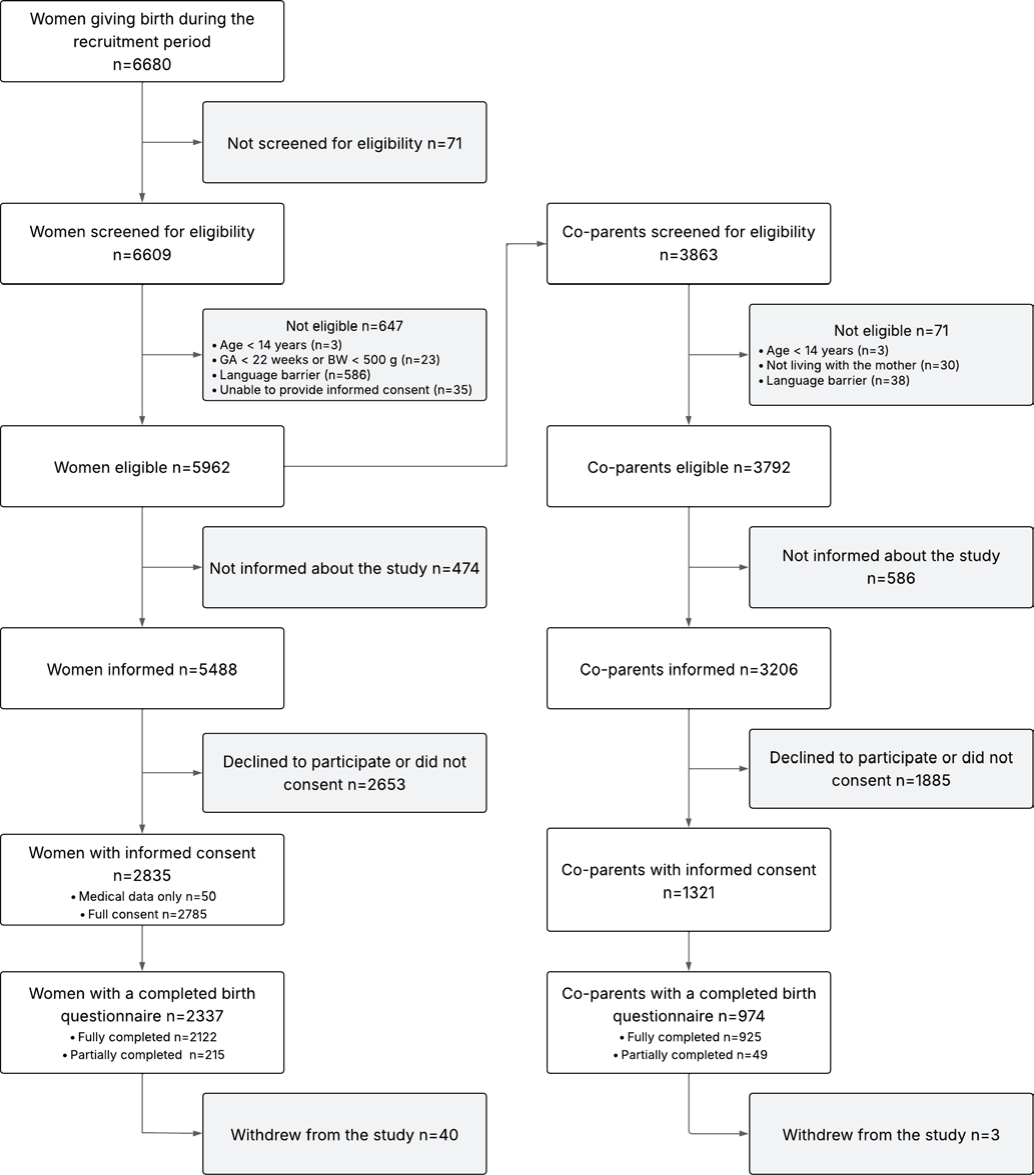
Flow chart of participant recruitment and inclusion in the SOCRATES cohort. CB-PTSD, childbirth-related post-traumatic stress disorder; SOCRATES, Stress Of Co-parents Related to A Traumatic Experience of birth across Switzerland.

Among 6680 women giving birth over the study period, 6609/6680 (98.9%) mothers were screened, and 5962/6609 (90.2%) were eligible (figure 2). The most common reason for ineligibility was language barriers (n=586/647; 90.6%). A total of 5488 mothers were informed about the study, 2835/5488 (51.7%) provided consent for extraction of clinical data, and 2785/5488 (50.7%) consented to complete the questionnaires. Of those, 2337/2785 (83.9%) started the baseline questionnaire, and 2122/2785 (76.2%) fully completed it. In parallel, 3863 co-parents were screened, 3792/3863 (98.2%) were eligible, 3206/3792 (84.5%) were informed, and 1321/3206 (41.2%) provided consent. Overall, 974/1321 co-parents (73.7%) started the baseline questionnaire, and 925/1321 (70.0%) fully completed it.

### Data collection

Data are collected from four complementary sources: (1) obstetric and neonatal clinical data from medical records, (2) self-administered online parental questionnaires, (3) a structured institutional survey completed by participating centres and (4) linkage with national hospital discharge datasets (Medical Statistics of Swiss Hospitals, which also include data from birth centres). Clinical data are recorded at baseline and include maternal medical and obstetrical history, pregnancy characteristics and complications, labour and childbirth management (eg, induction, analgesia, mode of childbirth), postpartum complications (eg, haemorrhage, infection), and neonatal outcomes such as Apgar score, birth weight and neonatal unit admission. Participants are invited to complete four online questionnaires, shortly after birth (baseline), and at 2, 6 and 12 months post partum. All questionnaires are provided in French, German, Italian and English. In parallel, a methodological collaboration was established with the Swiss-Peristat project (SNSF 222882), which aims to validate national perinatal care quality indicators. A subset of clinical data collected in SOCRATES will be used to estimate the accuracy of routinely collected hospital administrative data from the Swiss Federal Statistical Office.

### Outcome measures and instruments

Primary and secondary outcomes are measured using validated self-report instruments. The full list of instruments, respondents, domains and time points is detailed in table 1.

**Table 1:** Domains, validated instruments and timing of data collection for mothers and co-parents in the SOCRATES cohort

Domain	Topic	Validated scale	Number of items	Respondents and time points				Ref.
				Childbirth	2 months	6 months	12 months	
Childbirth	Fear of childbirth	Fear of Childbirth Visual Analogue Scale (FOCP-VAS)	1	◯	/	/	/	^54^
	Childbirth experience	Questionnaire for Assessing Childbirth Experience (QACE SF)	5	◉	/	/	/	^48^
	Traumatic experience	Peritraumatic Dissociative Experiences Questionnaire (PDEQ)	10	◉	/	/	/	^49^
	Satisfaction with care	Behaviour of the Mother’s Caregivers–Satisfaction Questionnaire (BMC-SQ)	7	◯	/	/	/	^50^
Physical health	General health	Minimum European Health Module (MEHM)	1	◉	/	/	/	^55^
		EuroQol Visual Analogue Scale (EQ-VAS)	1	◉	◉	◉	◉	^45^
	Early recovery	Obstetric Quality of Recovery-10 (ObsQoR-10)	10	◯	/	/	/	^56^
	Fatigue	Fatigue Assessment Scale (FAS), short selection at 6 and 12 months	10	/	◉	◉	◉	^38^
	Urinary incontinence	International Consultation on Incontinence Questionnaire–Urinary Incontinence (ICIQ-UI)	3	/	◯	◯	◯	^40^
	Anal incontinence	Jorge and Wexner anal incontinence score	3	/	◯	◯	◯	^41^
	Pain	Pain Intensity derived from the Brief Pain Inventory (BPI)	1	◯	◯	◯	◯	^39^
	Premenstrual symptoms	Premenstrual Symptoms Screening Tool (PSST)	1	◯	/	/	/	^57^
	Pelvic floor symptoms	Pelvic Organ Prolapse Distress Inventory-6 (PoPDI-6)	3	/	/	/	◯	^58^
Quality of life	Quality of life	EuroQol 5-Dimension 5-Level (EQ-5D-5L)	5	◉	◉	◉	◉	^45^
	Satisfaction With Life	Satisfaction With Life Scale (SWLS)	5	◉	/	/	◉	^59^
	Well-Being	The WHO–Five Well-Being Index (WHO-5 well-being)	5	◉	◉	◉	◉	^46^
	Flourishing	Secure Flourishing Index	12	/	/	/	◉	^60^
Mental health	Post-traumatic stress disorder (PTSD)	City Birth Trauma Scale for mothers/for partners (CB-PTSD)	29/29	/	◉	/	/	^25^ ^30^
	Depression, anxiety	Edinburgh Postnatal Depression Scale (EPDS and EPDS-3A)	10	◉	◉	◉	◉	^61^
	Stress	Perceived Stress Scale-4 (PSS-4)	4	◉	◉	◉	◉	^62^
	Loneliness	UCLA-Loneliness Scale-3 (UCLA-3)	3	◉	◉	◉	◉	^63^
	Resilience	Brief Resilience Coping Scale (BRCS)	4	◉	/	/	/	^64^
	Suicidal ideation	Patient Health Questionnaire-9 (PHQ-9) – item 9	1	◉	◉	◉	◉	^65^
Sexual health	Sexual function	Female Sexual Function-6 (FSFI-6)	6	/	/	◉	◉	^42^
	Sexual well-being	Natsal Sexual Well-being measure (NATSAL SW)	13	/	/	/	◉	^44^
Parenting	Bonding	Mother-to-Infant Bonding Scale (MIBS)	8	/	◉	/	/	^66^
	Parenting confidence	Me as a Parent Scale (MaaPs-SF)	4	/	◉	◉	◉	^67^
	Relationship	Brief Coparenting Relationship Scale (B-CRS)	14	/	/	◉	/	^68^
Support	Couple satisfaction	Couple Satisfaction Index (CSI-4)	4	◉	◉	◉	◉	^69^
	Social support	Multidimensional Scale of Perceived Social support (MSPSS)	12	◉	◉	◉	◉	^70^
Lifestyle	Sleep quality	Pittsburgh Sleep Quality Index (PSQI)	1	◉	◉	◉	◉	^71^
	Sleep	Insomnia Severity Index (ISI-3)	3	◉	◉	◉	◉	^72^
	Eating and body image	Maternal Body Image and Eating Behaviours Scale (MBIEB-S), short selection	4	/	◯	◯	◯	^73^
	Body image	Maternal Postpartum Quality of Life (MAPP-QOL)	1	/	◯	◯	◯	^74^
	Physical Activity	International Physical Activity Questionnaire (IPAQ short form)	2	◉	◉	◉	◉	^75^
	Physical Activity	Global physical activity questionnaire (GPAQ)	1	◉	◉	◉	◉	^76^
Work	Work-Family Conflict	Work-Family Conflict Scale (ISSP)	4	/	•	◉	◉	^77^

◯: mother; •: co-parent; ◉: Both mother and co-parent.

SOCRATES, Stress Of Co-parents Related to A Traumatic Experience of birth across Switzerland.

To address the primary objective (1), the prevalence and severity of CB-PTSD at 2 months post partum is measured using the City Birth Trauma Scale.^25^ This 29-item instrument, aligned with DSM-5 criteria, demonstrated excellent internal consistency and test–retest reliability in postpartum samples.^26 27^ A version specifically adapted for co-parents is used where applicable, featuring slightly modified wording to reflect their non-birthing role while preserving the instrument’s conceptual structure and psychometric properties.^28^,^31^

To address objective (2), depression and anxiety symptoms are assessed from the perinatal period to 12 months using the Edinburgh Postnatal Depression Scale (EPDS).^32^ This 10-item screening tool provides good internal consistency, is widely used in postpartum populations and validated in all four languages.^33 34^ In addition to the total score, anxiety symptoms are explored using the EPDS-3A subscale, which comprises items 3, 4 and 5, and has been proposed as a brief indicator of postnatal anxiety.^35^ Suicidal ideation is assessed using the ninth item of the Patient Health Questionnaire-9.^36^

In line with objective (3), maternal physical recovery is measured at baseline using the Obstetric Quality of Recovery-10,^37^ a 10-item scale scored from 0 to 10, with a total range from 0 to 100. It showed good internal consistency and test–retest reliability. Additional physical outcomes (ie, fatigue, Fatigue Assessment Scale),^38^ pain (using a single-item Numeric Rating Scale, 0–10), derived from the Brief Pain Inventory,^39^ urinary incontinence (International Consultation on Incontinence Questionnaire-Urinary Incontinence)^40^ and anal incontinence (Jorge and Wexner Score)^41^ are assessed longitudinally using short, validated tools with proven reliability in postpartum samples.

To address objective (4), maternal sexual health is assessed at 6 and 12 months post partum using the short version of the Female Sexual Function Index-6,^42^ which evaluates six domains (desire, arousal, lubrication, orgasm, satisfaction, pain). This instrument has demonstrated good to excellent internal consistency in postpartum populations.^43^ The Natsal Sexual Well-being Measure^44^ with good test–retest reliability is also administrated at 12 months post partum to assess broader sexual well-being.

For objective (5), health-related quality of life and well-being are assessed with the EuroQol 5-Dimension 5-Level questionnaire (EQ-5D-5L)^45^ and the WHO-5 Well-Being Index,^46^ both validated and widely used. The EQ-5D-5L is a generic measure of health status covering five dimensions (mobility, self-care, usual activities, pain/discomfort and anxiety/depression), each with five levels of severity, and includes a EuroQol Visual Analogue Scale ranging from 0 to 100 to assess overall self-rated health.^45^ The WHO-5 Well-Being Index is a five-item instrument assessing psychological well-being over the previous 2 weeks, with scores ranging from 0 to 100. It has demonstrated a good internal consistency and a robust unidimensional structure.^47^ At 6 months, an open-ended question invites mothers and co-parents to suggest, hypothetically, what could be implemented to improve parental well-being after childbirth.

Objective (6) focuses on the influence of clinical practices (eg, induction, mode of childbirth, perineal trauma), structural characteristics (eg, staffing levels, care protocols, availability of support services) and perceived care quality on health outcomes. Women’s birth experiences are assessed using four items from the Questionnaire for Assessing the Childbirth Experience.^48^ The Peritraumatic Dissociative Experiences Questionnaire^49^ assesses dissociative symptoms during labour, while the Behaviour of the Mother’s Caregivers Satisfaction Questionnaire^50^ evaluates relational aspects of care. Institutional-level characteristics derived from the maternity unit survey will be incorporated, including care models, staffing levels and workload indicators, organisational practices, availability of clinical protocols and support services and quality assurance procedures.

To meet objective (7), maternal and neonatal hospitalisations from 1 year before to 1 year after birth will be identified through deterministic and probabilistic linkage with the national Medical Statistics of Hospitals database, providing frequency, timing and diagnosis codes.

To address objective (8), we will examine how social determinants, gender roles and societal factors shape postpartum health and well-being. Individual-level measures include questions on perceived financial strain, employment conditions, experiences of discrimination, social support, paternity leave, division of domestic, caregiving tasks and mental load, and perceived gender inequities in the postpartum period.

In addition to validated instruments, the SOCRATES questionnaires include a set of ad hoc questions and adapted items designed to capture sociodemographic characteristics, family context and relevant behavioural and structural determinants of health. These items were selected based on their use in large-scale population surveys and were adapted when necessary to the perinatal context. Occupations and education levels are coded according to the International Standard Classification of Occupations (first two levels) and the International Standard Classification of Education-2011, respectively. Several items were drawn and/or adapted from national and international studies, including the Swiss Household Panel (eg, civil status, division of domestic tasks, health insurance), the French National Perinatal Survey (eg, birth plan, preference for epidural analgesia), the French ELFE cohort (eg, perceived partner support), the Irish MAMMI cohort (eg, factors affecting sexual life in the postpartum period) and the European Social Survey (eg, household income perception). An adapted version of the International Physical Activity Questionnaire was also included, following Swiss and WHO guidelines. All questionnaires were reviewed by healthcare professionals, patient associations and researchers, and pilot-tested for clarity, inclusiveness and cross-cultural relevance by a panel of young parents during the development phase.

In addition to individual-level data, the study collects institutional-level data through a structured survey administered to participating maternity units and birth centres. As no standardised instrument was available to comprehensively capture organisational and contextual characteristics of maternity care in Switzerland, a questionnaire was specifically developed for the SOCRATES study, building on previous national surveys and expert input. The development process included iterative discussions with multidisciplinary experts, item refinement and pilot testing to ensure clarity, relevance and feasibility across different care settings. The survey is completed once per institution by a senior staff member with detailed knowledge of local organisation (eg, head midwife, clinical expert or nursing manager). It comprises a comprehensive set of items covering structural characteristics (institution type, level of care, birth volume), models of care (including midwife-led care), infrastructure and clinical activity. It further collects detailed information on workforce capacity (full-time equivalents, vacancies, absences, skill mix, experience), organisation of work (shift systems, on-call arrangements, workload) and availability of services such as psychological support, breastfeeding support and social care. In addition, the questionnaire documents clinical practices and protocols (eg, monitoring, labour management, adherence to national or international guidelines), quality assurance activities, training opportunities and postpartum care pathways. These data enable multilevel analyses linking institutional characteristics with individual health outcomes and care experiences, and allow exploration of contextual determinants of postpartum health at the system level.

When validated versions were not available in all study languages, questionnaires were translated by professionals and subsequently reviewed by clinicians and researchers involved in the study, as well as by native-speaking users, to ensure clarity, relevance and appropriateness of wording across contexts. This process therefore followed a multistep, pragmatic approach, broadly aligned with established recommendations for the translation of health-related questionnaires in multinational studies.^51^ Minor adaptations were made where necessary to reflect the perinatal context. Where applicable, the psychometric properties of selected instruments will be assessed within the SOCRATES cohort, focusing on key measures used in primary analyses. These evaluations may include internal consistency and construct validity, depending on the specific instrument and planned analyses.

### Data management

Study data are collected and managed using Research Electronic Data Capture (REDCap), a secure, web-based software platform hosted on encrypted servers in Switzerland.^52 53^ The platform complies with Swiss federal data protection legislation and institutional security standards. Each participating family (mother and co-parent) is assigned a unique study identifier at the time of consent, allowing linkage of maternal and co-parent data while ensuring pseudonymisation of clinical and self-reported data prior to integration. REDCap provides structured case report forms with built-in logic checks and real-time validation rules to minimise entry errors.

Clinical data are entered into the electronic case report form by trained local research staff at each participating site. Questionnaires are completed online by participants via secure, individual access links. Access to identifiable information is restricted to authorised personnel at the local site level and to the central data management unit (DAUnit), in accordance with role-based access permissions. An audit trail automatically records all data manipulations, modifications, and exports. The REDCap platform also supports role-based access control, automated data exports to statistical software (R, Stata, SAS) and daily system backups. The DAUnit ensures system monitoring, compliance and recovery protocols. Quality assurance processes include periodic internal data control procedures, logic consistency checks and cross-validation of selected variables across data sources (eg, comparison between clinical records and self-reported data) to identify inconsistencies. Data from national hospital discharge records will be linked in accordance with the protocol approved by ethics committees and the Swiss Federal Data Protection Authority.

### Statistical analysis and weighing procedure

Descriptive analyses will be conducted to summarise participants’ characteristics and outcomes. Associations between predictors and outcomes will be examined using multivariable regression models, accounting for potential confounders identified through literature review, clinical expertise and directed acyclic graphs. Sensitivity analyses will be performed to evaluate the robustness of the findings. Missing items will be imputed using standard procedures whenever relevant (eg, multivariate imputation by chained equations). All analyses will be carried out using statistical software such as R or Stata.

To ensure representativeness at the national level, we will apply a weighting procedure that includes the following steps. First, base design weights are computed to account for differences in participation across maternity care institutions. Second, raking is applied to align the weighted sample distribution with known population margins, using key sociodemographic variables (education, income, age, Swiss nationality and parity). Predictive models (eg, Random Forest, XGBoost, Support Vector Machines) are employed to impute missing values in these key variables before calibration. Third, non-response adjustment weights are estimated through logistic regression using region, parental origin, education, ethnicity, age, living conditions, institution type and number of pregnancies as predictors. Predicted response probabilities from this model are then used to construct inverse probability weights, such that respondents with a lower predicted probability of response receive larger weights. To prevent undue influence of extreme weights, trimming is applied at predefined thresholds. The final adjusted weights are then assigned to all observations for subsequent analyses.

### Patient and public involvement

Patient and public partners were involved in the development of this study. The protocol was reviewed by *Santé sexuelle Suisse,* the Swiss umbrella organisation for sexual health counselling centres and sex education services, and by *Periparto*, a national non-profit organisation dedicated to perinatal mental health. Representatives from these organisations participated in the Steering Committee responsible for validating the study questionnaires. In addition, the questionnaires were pre-tested with a panel of young parents from diverse backgrounds, and revised based on their feedback to improve clarity, relevance and acceptability, especially for sensitive topics related to mental health, sexuality, parenting and family life. In response to this feedback, the questionnaires were refined to better capture the co-parent’s perceived role, time spent as a couple and time for oneself. During data collection, free-text comments from participants in the baseline questionnaire were also reviewed to identify recurrent concerns. These comments informed the development of follow-up questionnaires, including items on perceived pressure or judgement regarding parenting choices. This process helped ensure that participant perspectives were reflected in the content of the instruments. To support participants during data collection, each questionnaire includes a message encouraging those experiencing distress to seek help from their midwife or specialised support services. This includes the 24/7 anonymous helpline *Heart2Heart* (www.143.ch).

## Ethics and dissemination

The SOCRATES study is conducted in accordance with the Swiss Human Research Act and the Declaration of Helsinki. Ethical approval has been obtained from the relevant cantonal ethics committees (number 2024-02262). All participants provided informed consent prior to data collection. Additional separate consent forms were requested for data linkage with hospital discharge records and recontact for optional future follow-up. Confidentiality is ensured through strict pseudonymisation procedures and secure data management systems.

Dissemination will include original research articles across midwifery, obstetrics, psychiatry and public health disciplines, targeting high-impact, open-access journals. Findings will be presented at international and national conferences and shared through professional journals in French, German and Italian to enhance accessibility among midwives and other clinicians. A scientific communication project has been submitted for funding to disseminate the project’s results to the broader public, targeting especially parents and their close circles.
