# Supplementary material for: Health and well-being of mothers and co-parents during the first 12 months post partum: study protocol of the national SOCRATES cohort study in Switzerland
Source: BMJ Open. 2026 Jun 4;16(6):e121443. doi: 10.1136/bmjopen-2026-121443 (PMC13239364; doi:10.1136/bmjopen-2026-121443)
Supplement: Supplementary file 1 [file bmjopen-2026-121443.reviewer_comments.pdf]

PEER REVIEW HISTORY

BMJ Open publishes all reviews undertaken for accepted manuscripts. Reviewers are asked to complete a checklist review form and are provided with free text boxes to elaborate on their assessment. These free text comments are reproduced below.

ARTICLE DETAILS

Title (Provisional)

The health and well-being of mothers and co-parents during the first 12 months post-partum: study protocol of the national SOCRATES cohort study in Switzerland

Authors

Gaucher, Laurent; Desplanches, Thomas; Sormani, Jessica; Cattani, Gilles; Muller, Antonia N; Celetta, Émilienne; Widmer, Isabel N; Lorthe, Elsa; Grylka-Baeschlin, Susanne

---

VERSION 1 - REVIEW

**Reviewer**	**1**
**Name**	**Lim, Siew**
**Affiliation**	**Monash University, Health Systems and Equity**
**Date**	**04-May-2026**
**COI**	**None**

Thank you for the opportunity to review this manuscript on the protocol of a cohort study on the health and well being of mothers and co-parents in the first 12 months postpartum.

Introduction:

-In the first paragraph, it is unclear what clinical or public health problem this is aiming at solving. The primary study objective suggests that the key problem statement is on mental health, but this was not clear in the opening paragraph.

-There is some information on the evidence gap (e.g. mental health, quality of care), but these appeared as random picks, instead of being embedded in a context of problem statement. It is also not comprehensive framework to justify the list or types of variables proposed. An overarching rationale is needed here.

Study aims:

-some framework on how those variables relate to each other would be helpful

Data collection and outcome measures sections were clear and well developed.

Patient and public involvement:

It is commendable to have PPI in the design of this cohort. More details on Sante sexuelle Suisse and Periparto (are they patient and public advocacy groups?) and how they have been involved, will be helpful.

VERSION 1 - AUTHOR RESPONSE

Reviewer: 1

Dr. Siew Lim, Monash University

Comments to the Author:

Thank you for the opportunity to review this manuscript on the protocol of a cohort study on the health and well being of mothers and co-parents in the first 12 months postpartum.

We thank the reviewer for the careful reading of our manuscript and for these constructive comments.

Introduction:

-In the first paragraph, it is unclear what clinical or public health problem this is aiming at solving. The primary study objective suggests that the key problem statement is on mental health, but this was not clear in the opening paragraph.

-There is some information on the evidence gap (e.g. mental health, quality of care), but these appeared as random picks, instead of being embedded in a context of problem statement. It is also not comprehensive framework to justify the list or types of variables proposed. An overarching rationale is needed here.

We thank the reviewer for these important comments. We agree that the initial introduction did not sufficiently articulate the clinical and public health problem addressed by the study, nor the overarching rationale linking the different domains investigated.

We have substantially revised the introduction to address these points. First, we now frame the first year after childbirth as a critical yet under-addressed period for parental health and family well-being, characterised by unresolved health problems that may extend beyond the early postpartum weeks. Second, we introduced an explicit conceptual rationale based on the biopsychosocial model, social determinants of health and the WHO framework for quality of maternal and newborn care. This positions postpartum health as a multidimensional process shaped by mental, physical, sexual, social, clinical and structural determinants.

We also reorganised the introduction so that mental health and care experiences are presented as key components of this broader framework rather than as isolated evidence gaps. Finally, we added a conceptual framework (Figure 1) to illustrate the hypothesised relationships between determinants, childbirth-related exposures and postpartum health and well-being trajectories, complementing Table 1, which details the measured variables, instruments and time points.

Study aims:

-some framework on how those variables relate to each other would be helpful

We thank the reviewer for this helpful suggestion. We have now added a conceptual framework (Figure 1) to clarify the hypothesised relationships between social determinants, pregnancy and childbirth-related exposures, and postpartum health and well-being trajectories. This framework is informed by established models, including the biopsychosocial model (Engel), social determinants of health frameworks (WHO), and the WHO framework for quality of maternal and newborn care (Tunçalp et al.). It is intended to complement Table 1, which details the measured variables, instruments and time points.

Data collection and outcome measures sections were clear and well developed.

We thank the reviewer for this positive feedback.

Patient and public involvement:

It is commendable to have PPI in the design of this cohort. More details on Sante sexuelle Suisse and Periparto (are they patient and public advocacy groups?) and how they have been involved, will be helpful.

In line with this relevant suggestion, we have revised the "Patient and Public Involvement" section to better describe the organisations involved and their contributions. We now specify that *Santé sexuelle Suisse* is the Swiss umbrella organisation for sexual health counselling centres and sex education services, and that *Periparto* is a national non-profit organisation dedicated to perinatal mental health. We also clarified how these organisations contributed to the study, including protocol review, and participation in the Steering Committee. In addition, we expanded the section to describe how feedback from young parents and free-text comments from participants informed refinements to subsequent questionnaires, including items on co-parent role, couple time, personal time, and perceived pressure or judgement about parenting choices.

Reviewer: 1

If you have selected 'Yes' above, please provide details of any competing interests.: Not applicable

All authors declare that they have no competing interests.
